# B1.12: a novel peptide interacting with the extracellular loop of the EBV oncoprotein LMP1

**DOI:** 10.1038/s41598-019-39732-y

**Published:** 2019-03-13

**Authors:** Nihel Ammous-Boukhris, Amor Mosbah, Wajdi Ayadi, Emna Sahli, Soizic Chevance, Arnaud Bondon, Ali Gargouri, Michele Baudy-Floc’h, Raja Mokdad-Gargouri

**Affiliations:** 1 Center of Biotechnology of Sfax, Laboratory: Molecular Biotechnology of Eukaryotes, University of Sfax, Sfax, Tunisia; 2 BVBGR-LR 11ES31, ISBST, University of Manouba, Biotechnopole Sidi Thabet, 2020 Ariana, Tunisia; 3 COrInt, ISCR UMR CNRS 6226, Université de Rennes 1, Rennes, France; 4 Plate-forme PRISM, Biosit, SFR UMS CNRS 3480 - INSERM 018, Université de Rennes 1, Rennes, France; 5 Center of Biotechnology of Sfax, Plate-forme of Analysis, University of Sfax, Sfax, Tunisia

## Abstract

Latent membrane protein 1 (LMP1) encoded by the Epstein-Barr virus (EBV) plays an important role in EBV-induced cell transformation. Down-regulation of the LMP1 expression had shown promising results on cancer cell therapy. In this study, we identified by Phage display a novel peptide called B1.12 (ACPLDLRSPCG) which selectively binds to the extracellular loop (B1) of the LMP1 oncoprotein as demonstrated by molecular docking, NMR and ITC. Using an LMP1 expressing cell line, we showed that B1.12 decreased cell viability, and induced G0/G1 cell cycle arrest. In addition, the expression of A20, pAkt, and pNFkb (pRelA536) in C666-1 cells treated with B1.12 decreased compared to the untreated cells. In conclusion, we selected a novel peptide able to bind specifically to the extracellular loop of LMP1 and thus modulate its oncogenic properties.

## Introduction

Phage display is a powerful technique that allows the presentation of multiple peptides on the surface of filamentous phage particles for diverse applications^1^. Recently, the bio-panning of phage display peptide library on whole cells was proven successful for selecting peptides targeting cancer cells^2,3^. Zhou *et al*. have identified a tumor cell-binding peptide able to control ovarian cancer cell viability, migration, invasion and adhesion *in vitro* as well as tumor growth and metastasis *in vivo*^4^. Furthermore, Ma *et al*. used a substractive whole cell bio-panning to identify peptides that bind specifically to the membrane of human esophageal squamous cell carcinoma (ESCC). These peptides are very useful for imaging, detection and targeted therapy^5^. In the present study, we focused on the latent membrane protein 1 (LMP1) which is the major oncoprotein of the Epstein Barr Virus (EBV) that is over-expressed in some EBV-associated malignancies such as nasopharyngeal carcinoma (NPC)^6^. Structurally, LMP1 is an integral membrane protein consisting of a short cytoplasmic N-terminus region of 20 amino acids, a trans-membrane domain with six membrane-spanning segments that anchor LMP1 in the plasma membrane, and a long cytoplasmic C-terminal region of 200 amino acids^7^. The cytoplasmic region contains transformation effector sites (TES-1 and TES-2), which are similar to the tumor necrosis factor (TNF) receptor signaling regions and regulate cellular signaling pathways^8–12^. Previous studies have indicated that tumors displaying high levels of LMP1 tend to be more malignant with frequent distant metastasis^8^. Therefore, it is important to reduce the expression level of this oncoprotein to slow tumor progression and invasion, and to improve the response to treatment and thus patient survival. In this context, different approaches had been developed based on the use of DNAzyme, specific antibodies and siRNA^13–16^. In the present work, we used phage display approach to select peptides targeting the first extracellular loop of LMP1. It is important to mention that to date, there is not a known ligand interacting with LMP1 and that this receptor is activated constitutively^12^. With this in mind, we thought that the search for specific peptides targeting the extracellular LMP1 loops can probably mimic a ligand that could prevent the activation of LMP1 and consequently the signaling cascade controlled by this oncoprotein. Therefore, we synthesized a peptide similar to the sequence of LMP1’s first extracellular loop to use it as a target in the bio-panning of the phage library. Usually, the panning is performed using proteins or whole cells: in the present work, we used a synthetic peptide as a target to screen the phage library. After three rounds of panning, we selected 15 phage clones that have binding capacity to the target. Among them 6 were sequenced and we retained the peptide B1.12 that can strongly bind to the B1 loop of LMP1. Furthermore, we characterized the interaction between the target B1 and the selected peptide B1.12, and studied the effects of this peptide on regulating the cell cycle, apoptosis, and the PIK3/Akt signaling pathway using cellular models.

## Results

### Identification of phage peptides that specifically bind to the LMP1 loop

**Figure 1:**
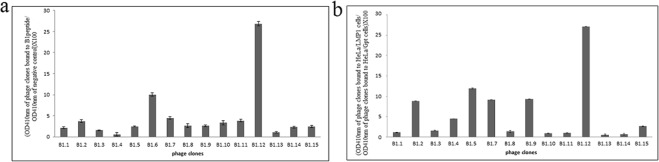
ELISA performed with 15 individual phage clones randomly picked up from the eluted phage pool after the 3rd round of bio-panning. The B1 peptide (**a**) or HeLa cell lines (**b**), were coated and 10^10^ pfu of each phage were added. Blocking buffer without B1 peptide was used as negative control for (**a**), and HeLa/Gpt cells were used as negative control for (**b**). Data represent the binding of the phage clones to the B1 target (**a**) and HeLa cells (**b**) as the mean ± SD of three separate experiments.

Using the phage display technology, we identified peptides that specifically bind to the extracellular loop of LMP1. Firstly, we synthesized the B1 peptide based on the sequence of the first extracellular loop of LMP1 which served to screen the peptide library (Ph.D.-C7C). The Ph.D.-C7C is a combinatorial library of random 7-mer peptides based on N-terminal fusion to the coat protein pIII of M13 phage. The randomized segment is flanked by a pair of cysteine residues (ACXXXXXXXCGGGS-pIII). After each of the three rounds of bio-panning, the input/output phage ratio was calculated. The data showed that the number of the third round phages was 166 times higher than that of the first round (Table 1). ELISA was performed to confirm the binding affinity of 15 randomly selected phage clones to the target B1 and HeLa cell line. Of the 15 phage clones tested by ELISA, 6 were selected and sequenced. The deduced peptide sequences were analyzed and alignment analyses did not reveal homology between the different peptide sequences. Among these clones, we retained the B1.12 that showed the highest binding affinity on both cell lines and peptide B1 (Fig. 1a,b).

**Table 1:** Selective enrichment of phage clones from phage display C-7-C library during panning.

phd library	1st round	2nd round	3rd round
Phage input (cfu)	2.00E + 12	2.00E + 11	1.00E + 11
Phage output (cfu)	3.10E + 05	3.00E + 06	5.00E + 07
Recovery rate	1.55E − 07	1.50E − 05	5.00E − 04

### Peptide synthesis and analysis of the B1/B1.12 interaction

**Figure 2:**
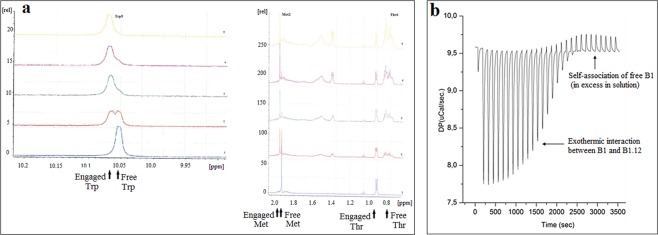
Chemical shift changes observed in NMR experiment (**a**). Spectrum from 1 to 5 indicate the different proportions of B1:B1.12 used in titration experiment (1:1; 3:1; 5:1; 7:1; 9:1, respectively). Arrows indicate the engaged and free amino-acids before and after B1/B1.12 interaction. SI: Signal Intensity. ITC profile showing the interaction between the target B1 and the selected peptide B1.12 (**b**). The figure shows the evolution of the interaction (Exothermic) until all the B1 binding sites were saturated and self-association of B1.12 occurs.

All peptides: B1 (CMSDWTGG), B1.12 (ACPLDLRSPCG), and B4 (ACNTGSPYECG) were synthesized using standard solid-phase Fmoc chemistry. N-terminus FITC labeled B1.12 was also synthesized. The purity of the peptides was assessed by HPLC to >95% (Supplementary Fig. S1). By mass spectrometry, the experimental mass-to-charge (*m*/*z*) ratios measured for all peptides were as expected (856.9 M, and 1132.2 M, and 1100.41 respectively). These peptides were used to investigate the interaction target/selected peptide using NMR and ITC. As shown on Fig. 2a,b, B1.12 peptide bound specifically to its target B1. Interestingly, on the basis of the NMR spectrum, the interaction between the two peptides can be detected through the chemical shift variation of three amino acids of the B1 peptide which are Met_2_, Trp_5_ and Thr_6_ residues. Besides, we noticed a conformational variation in the engaged molecule (B1) that is due to the variation in the chemical environment (Fig. 2a). On the other hand, ITC profile confirmed the binding of the B1.12 peptide to its target B1 peptide at 30 °C (Fig. 2b). Aliquots of 2 μL of B1 peptide (0.2 mM) were repeatedly injected into the sample cell containing B1.12 peptide (0.01 mM). As evident from the titration profile, the exothermic heat flow decreases with the increase of the number of injections until saturation was reached. The exothermic binding reaction essentially ceases after nineteen injections when all of the peptide B1.12 molecules present in the cell were bound to the B1 peptide molecules, and the addition of the first peptide causes no more heat flow.

### Molecular Docking

**Figure 3:**
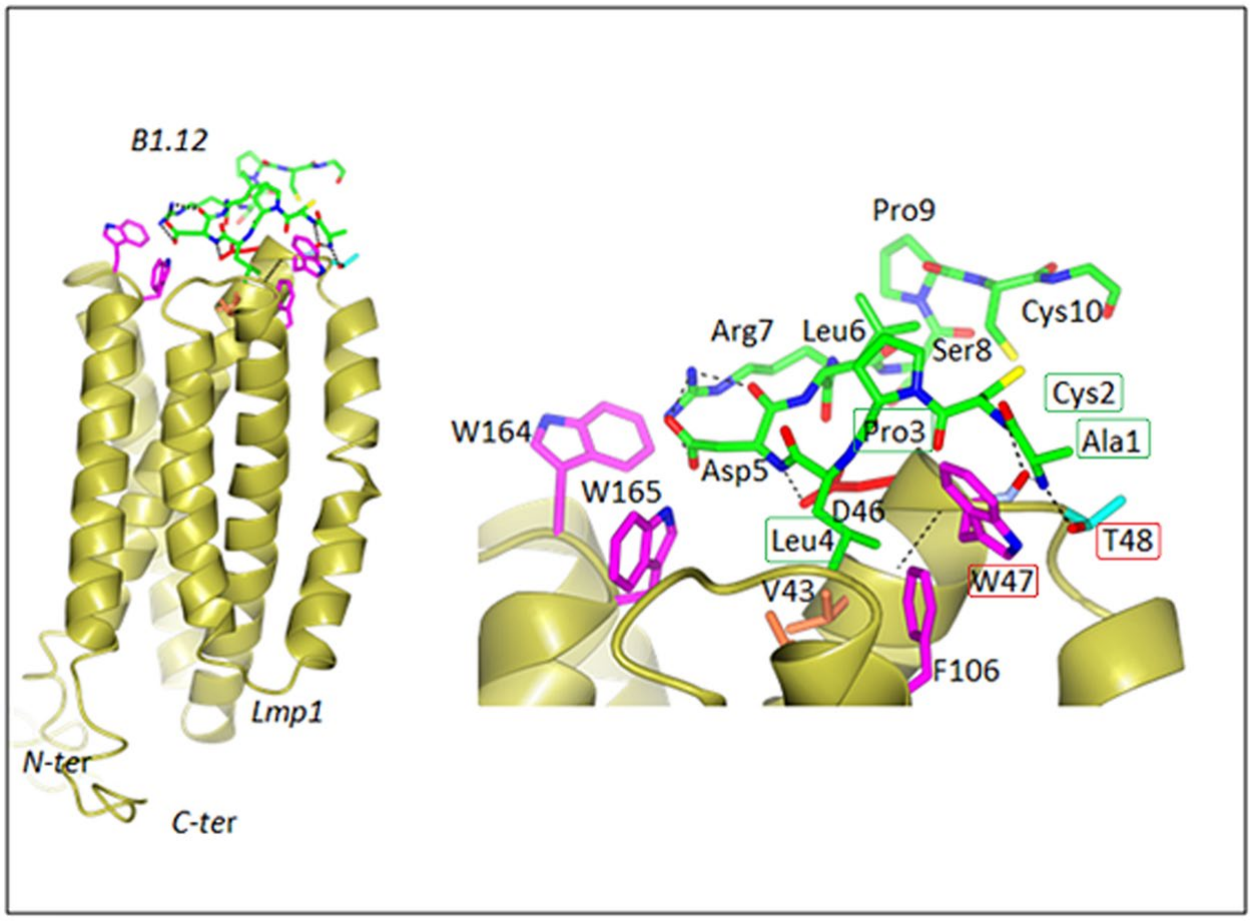
Molecular Docking of the N-terminal domain of LMP1 with the B1.12 peptide. LMP1 model is shown as a yellow ribbon and the B1.12 peptide is shown as a line model. The carbon, oxygen, and nitrogen atoms are colored in green, red, and blue, respectively. Amino acids involved in the interaction are framed in the figure in red for LMP1’s amino acids, and in green for B1.12 amino acids.

To further validate the binding of the B1.12 peptide to its target, we carried out a docking simulation analysis using the PatchDock and Fire Dock programs. On the basis of global energy amount, we selected the more suitable results. Interaction sites among the interface residues of both the N-terminal region of LMP1 and B1.12 peptide were chased by PSAIA. The model shows that the N-terminal region of LMP1 binds specifically to B1.12 peptide through interactions involving 3 pairs of amino acids (Fig. 3). All involved interactions were either Van der Walls or polar (Table 2). Furthermore, interaction studies on mutants of the ligand affecting the aa required for the peptide-ligand binding as discovered by NMR, have shown that the overall interaction energy increases when the Trp is mutated to Ala, attesting the specificity of our selected peptide (WT: −49.82 compared to −45.7 for the mutated model, Table 3). However, by changing the Thr to Ala, the overall energy of the interaction decreases considerably (Table 3). These results suggest that Trp is critical to maintain the binding B1.12/LMP1, but Thr is of less importance for this interaction.

**Table 2:** Interaction types evaluated by Molecular docking between the N-terminal region of LMP1 and the B1.12 peptide.

A 47 TRP	B 1 ALA	vanDerWaals
A 47 TRP	B 2 CYS	Polar, vanDerWaals
A 47 TRP	B 3 PRO	Polar, vanDerWaals
A 47 TRP	B 4 LEU	vanDerWaals
A 48 THR	B 1 ALA	vanDerWaals

A: aa residues of LMP1, B: aa residues of B1.12 peptide.

**Table 3:** Global energy evaluation for the interactions of wtLMP1, Mutant1-LMP1, and Mutant2-LMP1 with the B1.12 peptides, respectively.

	Wild type LMP1	Mutant 1: Trp to Ala	Mutant 2: Thr to Ala
Global Energy	−49.82	−45.70	−70.39

The results shown in the table indicate that wtLMP1 binds more effectively to the B1.12 peptide which proves its specificity.

### Competitive inhibition test for peptide binding

**Figure 4:**
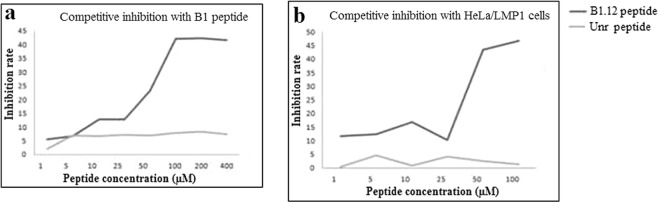
Competitive inhibition of the binding of the phage clone B1.12 by its corresponding peptide. This experiment was done with 2 targets: (**a**) B1 peptide, and (**b**) HeLa/LMP1 cells. The average inhibition rates at different concentrations of the peptide are shown. Independent experiments were repeated three times.

The competitive inhibition test was performed to determine whether the peptide B1.12 and its corresponding phage clone could compete for the same binding site of the target B1 on both HeLa/LMP1 cells as well as the synthetic peptide itself. We demonstrated that with an increase in the concentration of B1.12, the inhibition ratio increased gradually. When the peptide concentration was 400 µM, the inhibition ratio was approximately 43% (Fig. 4a). The unrelated peptide had no significant effect on the binding of positive phage B1.12 to the target B1. Furthermore, competitive inhibition assay was conducted on HeLa cells expressing or not LMP1. Our data showed that the inhibition ratio reached 45% when the peptide concentration is 100 µM (Fig. 4b).

### B1.12 affects cell viability of NPC cell line *C666.1*

**Figure 5:**
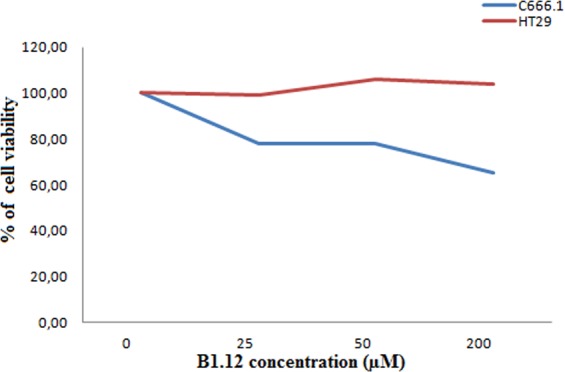
The cell viability of C666.1 cells treated with different concentrations of B1.12 peptide for 48 hours, and then assessed by MTT. C666.1 cells were incubated with various concentrations (0, 25, 50, and 200 μM) of B1.12 peptide, and cell viability was assessed after 48 h by MTT assay. The cell viability was expressed as a percentage of the viability against untreated cells used as control: % of Viability = (OD590 nm of treated cells/OD590 nm of untreated cells) × 100.

To examine whether the peptide B1.12 affects cell viability, we performed MTT assay using C666.1 cell line expressing LMP1. We showed that the viability of the cells gradually decreases with increasing peptide concentration to about 60% in the presence of 200 µM of B1.12. However, the viability of the control cells (HT129) was maintained at 100% in the presence of different concentrations of the peptide B1.12 (Fig. 5).

### Cellular uptake studies

**Figure 6:**
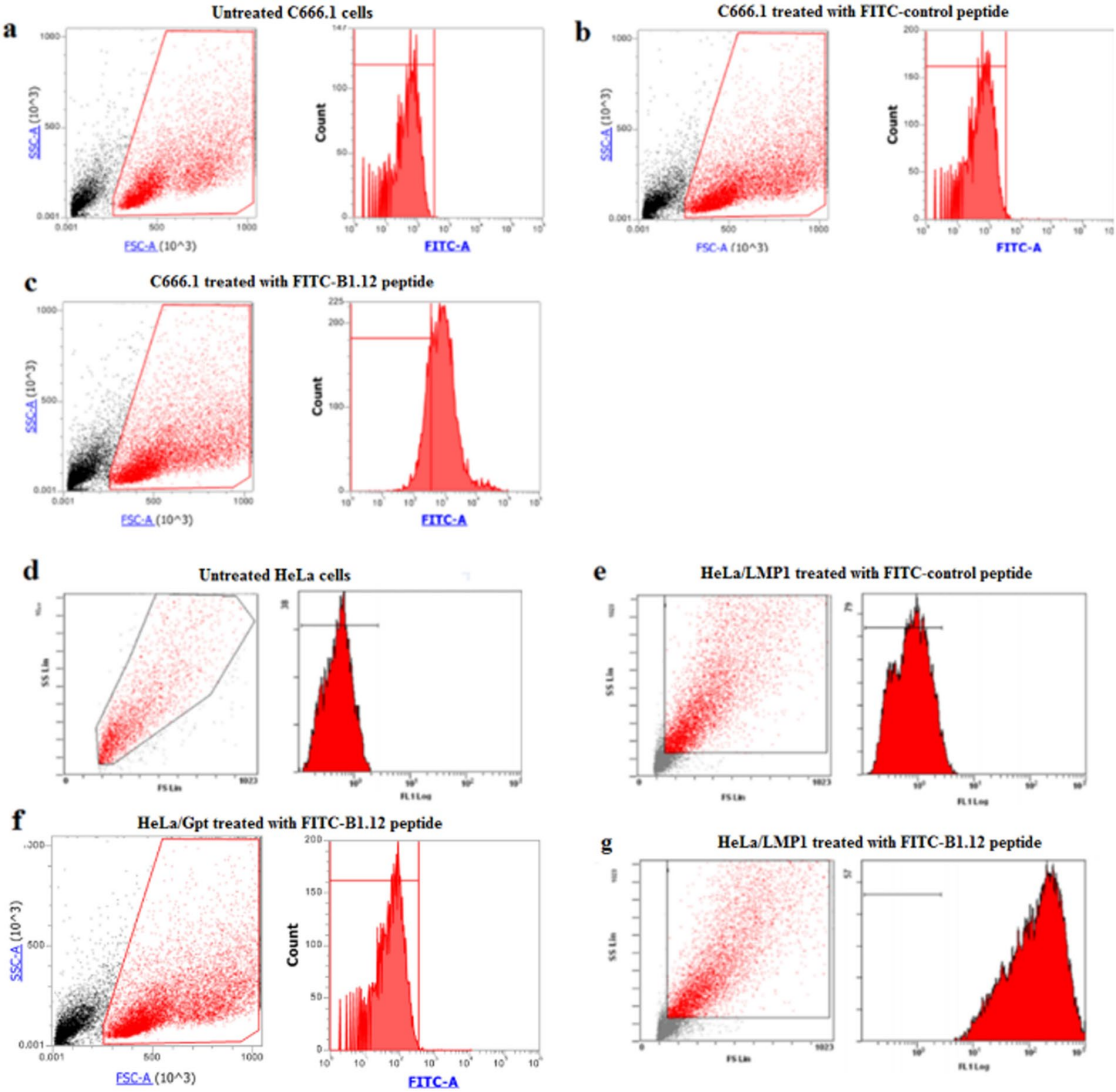
The cellular uptake of FITC-labeled B1.12 by C666.1, and HeLa cells. Panels a, b, and c show flow cytometric histograms of C666.1 cells untreated (**a**), treated with FITC-labeled control peptide (**b**), or with FITC-labeled B1.12 peptide (**c**). Panels d, e, f, and g represent flow cytometric histograms of HeLa cells. (**d**) Untreated HeLa cells, (**e**) HeLa/LMP1 cells treated with FITC-labeled control peptide, (**f**) HeLa/Gpt cells treated with FITC-labeled B1.12 peptide, and (**g**) HeLa/LMP1 cells treated with FITC-labeled B1.12 peptide.

The uptake efficiencies of FITC-labeled B1.12 peptide in C666.1, and HeLa cells were quantitatively measured by the flow cytometric analysis. The control-FITC labeled peptide had low nonspecific uptake in both cells (Fig. 6b,e). However, the B1.12 peptide exhibited significantly higher uptake (P = 0.0002) compared to the unrelated peptide in LMP1 positive cells (Fig. 6c,g). In LMP1 negative cells (HeLa/Gpt), no binding of the FITC-labeled B1.12 peptide was noticed (Fig. 6f).

**Figure 7:**
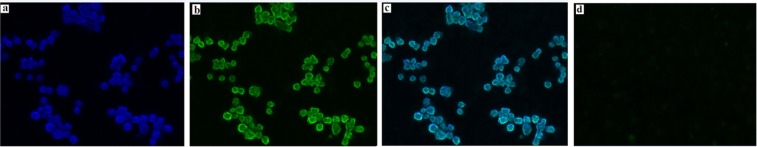
Immunofluorescence imaging of C666.1 cells. Log phase cells were fixed and stained for (**a**) LMP1 detection (blue) and (**b**) 10 µM FITC-labeled B1.12 (green). The merged images demonstrate colocalization of B1.12 and anti-LMP1 antibody signals (**c**). HeLa/Gpt cells treated with 10 µM FITC-labeled B1.12 served as negative control (**d**).

Furthermore, to confirm the specific binding of B1.12 to C666.1 cells, we performed fluorescence microscopy assay using FITC-labeled peptide B1.12. First, we localized the LMP1 expression using specific antibody in C666.1 cells (Fig. 7a). Then, we visualized the binding of B1.12 after incubating the cells for 2 hours with 10 µM of FITC-labeled B1.12 peptide (Fig. 7b). Most FTIC-labeled peptide (green) co-localized with LMP1 (blue), which means that B1.12 is specific to LMP1 (Fig. 7c). To confirm the B1.12 specificity, we performed HeLa/Gpt cells treatment with 10 µM of FITC-labeled B1.12 peptide. As presented in Fig. 7d, no binding was observed.

### Effect of B1.12 on cell cycle

**Figure 8:**
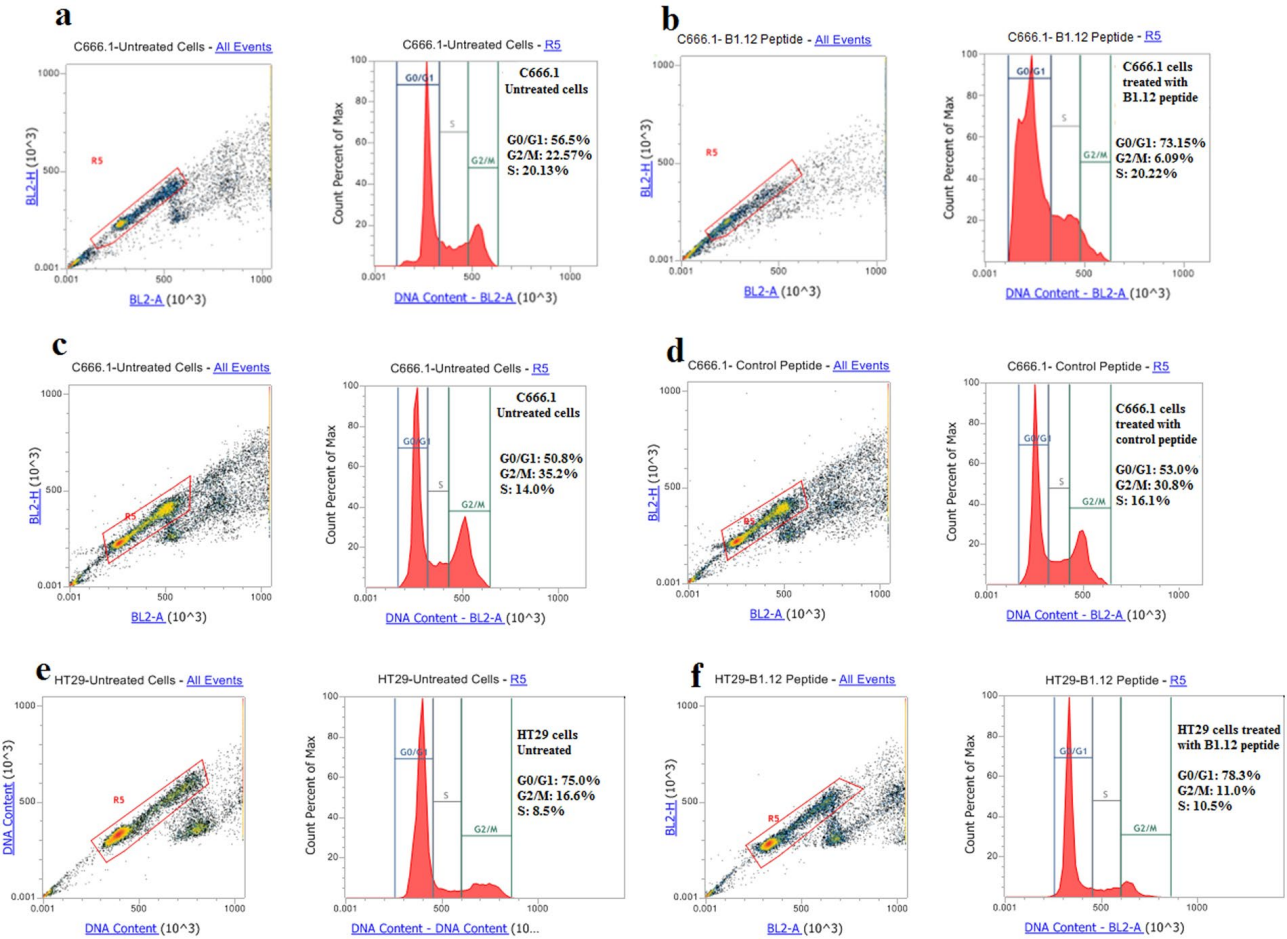
Effects of B1.12 treatment on the cell cycle. C666.1 cells were treated for 24 hours with (**b**) 50 µM B1.12 peptide, (**d**) 50 µM control peptide, then fixed, stained with propidium iodide and analyzed for DNA content by FACS. Similarly, HT29 cells were treated with B1.12 peptide at the same conditions (**f**). The untreated cells served as the negative control (**a**,**c** and **e**). The percentage of cells in each cell cycle phase was determined and shown for each sample.

It is well known that LMP1 promote cell growth and activate cell cycle progression, therefore, we studied the effect of B1.12 and control peptide on cell cycle progression of the NPC-derived cell line C666.1 and on HT29 cells. After treatment with 50 µM of B1.12, 73% of the cells were blocked at G0/G1 phase compared to 56% for the untreated cells (Fig. 8a,b). Furthermore, when treating with 50 µM of control peptide (Fig. 8c,d), no difference in the C666-1 cell cycle progression was seen compared to the untreated cells (53% vs 50.8%). On the other hand, we tested the effect of B1.12 on a control cell line (HT29). Our results showed that the cell cycle phases still unchanged (78.3% vs 75%) with or without B1.12 treatment (Fig. 8e,f).

### Effect of B1.12 on LMP1-dependent pathways

**Figure 9:**
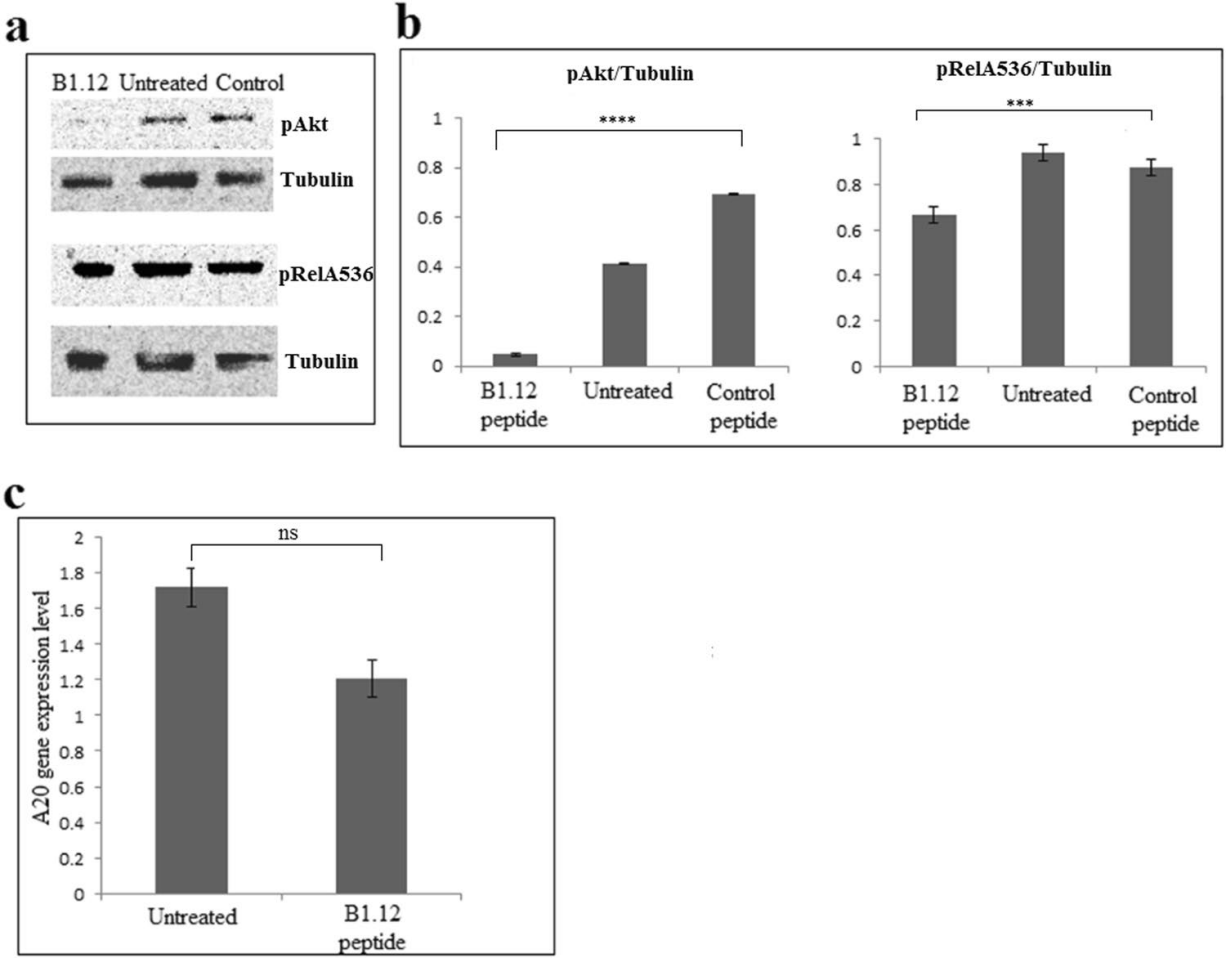
Western blot analysis of pAkt and pRelA/536 expression, in C666.1 cell line treated or not with 100 µM of B1.12 peptide or with 100 µM control peptide (**a**). Histograms representing the ratios pAkt/Tubulin and pRelA536/Tubulin expression are shown in (**b**). Density quantification of each band was assessed by the ‘image-j’ software. Data are represented as mean ± SD (n = 4 biological replicates), and P-value was evaluated by one-way ANOVA test, ***P = 0.0006, and ****P < 0.0001. Expression level of A20 in both B1.12-treated and untreated C666.1 cells are represented in (**c**). Data are represented as mean ± SD (n = 3 biological replicates), and P-value was evaluated by two-tailed Mann-Whitney *u* test (P = 0.1). ns: non significant.

To examine whether the binding of B1.12 to LMP1 affects the LMP1-mediated signaling pathways, we focused on the expression of pAkt and pNFκb (pRelA536) proteins in C666.1 cells treated or not with B1.12 peptide. Western blot showed that the expression of pAkt is low in B1.12-treated-C666.1 compared to those in the untreated or treated with control peptide (Fig. 9a). Furthermore, western blot showed a weak change in the pNFκb (pRelA536) expression with B1.12 in comparison with the untreated C666.1 or treated with control peptide (Fig. 9a). Histograms showing the ratios of pAkt/Tubulin and pRelA536/Tubulin were presented in Fig. 9b. Moreover, we investigated the expression level of A20 which is a well-known LMP1-target in C666.1-B1.12 treated and untreated cells. Interestingly, in the C666.1-B1.12 cells, the expression of A20 decreased compared to untreated cells indicating that B1.12 treatment affected LMP1 signaling pathway (Fig. 9c).

## Discussion

NPC is highly prevalent in some regions including southern China and Southeast Asia. EBV infection is constantly associated with NPC as indicated by the presence of viral transcripts and protein antigens in tumor cells^17^. EBV is a lymphotropic human gamma herpes virus that has been implicated in the pathogenesis of several human malignancies including Burkitt and Hodgkin lymphomas, NPC and gastric carcinoma^18^. Among the EBV proteins expressed in NPC cells, LMP1 exhibits properties of a classical oncoprotein. It activates different signaling pathways, such as nuclear factor kappa B (NFκB), c-jun N-terminal kinases (JNK), mitogen-activated protein kinases (MAPK), Janus kinase (JAK), serine-threonine protein kinase (Akt), activators of transcription protein (STAT), and others involved in the proliferation, apoptosis, and metastasis of tumor cells^19,20^. Therefore, LMP1 is considered as the primary oncogene of the EBV and thus a potential target for NPC targeted therapy^21^. It has been shown that down-regulation of LMP1 expression using RNA interference is potentially effective in the prevention of metastatic NPC and the reduction of NPC radio-resistance. Furthermore, Yang *et al*. designed specific DNAzymes (synthetic, single-stranded DNA catalysts) that can bind and cleave the LMP1 mRNA resulting in suppressing the expression of LMP1 and affecting LMP1-associated signaling pathways^18^. On the other hand, inhibition of LMP1 expression by siRNA in LMP1 positive NPC derived cell line C666-1, lead to an arrest of the cell cycle and increased the sensitivity of the cells to cisplatin^16^.

Phage display technology has been extensively used to identify small molecules, such as antibodies or peptides that bind to specific targets^4,22^. Regarding their properties such as high efficiency in cell-penetrating and strong biding efficacy with low immunogenicity, peptides are good candidates for clinical diagnosis and treatment of various types of cancers^1,2^. Indeed, several works described peptides selected by phage display, and targeting tumor cells. In breast cancer, a αFGF-binding peptide, AP8, isolated by phage display technology, provided an effective αFGF/FGFRs antagonist that might have potential applications in the treatment of breast cancer, characterized by the upregulation of αFGF/FGFRs^23^. Furthermore, Wang *et al*. identified a novel peptide, (NPMIRRQ), targeting cells and specifically ovarian cancer tissuesthatcan be applied in the diagnosis and treatment of ovarian cancer^24^. A novel peptide GP-5, isolated by whole-cell subtractive panning of the phage displayed 12-mer peptide library, has also been selected and binds specifically to gastric cancer (GC) cells and tissues. This peptide may thus be used in the molecular imaging for detection and/or in targeted drug delivery, in combination with other biological or chemical agents^25^.

In this work, we used synthetic peptides as target to screen a C-7-C phage display peptide library to identify peptides that specifically bind to the external loop of LMP1. After three rounds of panning, many phage candidates showed strong binding affinity to the target. Among them, the B1.12 phage which bound most effectively to its target as demonstrated by NMR experiments. NMR data showed also that B1.12 peptide interacts with three residues Met^2^, Trp^5^ and Thr^6^ of the B1 target peptide. Interestingly, almost same residues were involved in the interaction region between the N-terminal domain of LMP1 and the B1.12 peptide investigated by the docking prediction tools.

Further, we used two cell lines namely C666.1 and HeLa/LMP1 to prove the specific binding of the selected B1.12 peptide. For functional studies, we retained C666.1 which is an NPC tumor derived-cell line that maintains LMP1 expression and thus more suitable for testing the inhibitory effect of our peptide. We showed that the cell viability gradually decreased with increasing peptide concentration to about 60% in the presence of 200 µM of B1.12, whereas, the viability of the control cells (HT129) was maintained at about 100% in the presence of different concentrations of the peptide B1.12.

Besides, we demonstrated by flow cytometry, that B1.12 decreases cell proliferation by inducing G0/G1 cell cycle arrest of C666.1 treated cells (73% compared to 56% for untreated cells).

On the other hand, we tested the effect of B1.12 treatment on LMP1-involved signaling pathways. For example, we investigated the expression of pAkt and pNFκb (pRelA536) in C666.1 cells after incubation with the peptide B1.12. We noticed that, the expression of both pAkt and pRelA536 decreased in B1.12-treated-C666.1 compared to those in the untreated or treated with control peptide more markedly for pAkt, suggesting that LMP1 could modulate some signaling pathways through its membrane domain. Indeed, previous studies have shown that LMP1 transmembrane domains interacts to activate NFκB pathway in HEK293 LMP1-transfected cells^26^. In addition, Pratt *et al*., showed that the involvement of the carboxy-terminal region of LMP1 in the apoptosis regulation of B cells is directly linked to the activity of its six trans-membrane domains^27^.

It is well established that LMP1 induces A20 (TNFAIP3) expression^28^, therefore, we studied the expression level of this gene in C666.1-B1.12 treated and untreated cells. Our data showed that the A20 level decreased in the C666.1 cells after B1.12 treatment confirming that B1.12 modulates LMP1 activity and affects its role in cell signaling pathways especially the NFκB pathway.

In summary, we have identified a novel peptide which specifically binds to the extracellular loop of LMP1 and modulates its oncogenic effect by acting probably as an antagonistic ligand. It is evidently warranted that the B1.12 peptide specifically targets LMP1 expressing cells which provide possibilities for using it in imaging, detection, and targeted therapy. Further studies on animal models holding tumor cells expressing the viral oncoprotein LMP1 are obviously needed to confirm our data.

## Methods

### Cell lines and culture

Three cell lines were used: C666-1 which is a native EBV-infected NPC-derived cell line constitutively expressing LMP1, HeLa cells transfected with native vector (Gpt) or harboring the cDNA encoding LMP1, and HT-29 which is a human colon cancer cell line All cells were maintained in RPMI medium 1640 supplemented with 5% (V/V) FBS (Gibco), 4 µg/mL Gentamycine and 2 mM L-glutamine at 37 °C in a humidified atmosphere of 5% CO_2_. These cell lines were kindly provided by Pr P. Busson (IGR-France).

### Selective Phage enrichment

Peptide harboring phage selection was performed as described below. A solution of 1 mg/mL of the B1 peptide in 100 mM NaHCO_3_ (pH 8.6) was prepared and 100 μL was coated on a 96-well plate and incubated overnight at 4 °C. The coating solution was poured off and blocking buffer (skimmed milk at 5 mg/mL in 100 mM NaHCO_3_) was added for 1 hour at 4 °C. After washing with 0.1% (v/v) Tween-20/TBS, 10 μL (2 × 10^11^ plaque forming units, pfu) of original library, Ph.D™-C7C Phage Display Peptide (New England Biolabs), were diluted in 100 μL TBST and added to the plate well for 2 hours at room temperature with gentle agitation. After ten washes with 0.1% TBST, the bound phages were eluted with 0.2 M glycine-HCl (pH 2.2) and neutralized with 1 M Tris-HCl (pH 9.1). Eluted phages were amplified in 20 mL LB inoculated with the *E. coli* ER2738 strain, purified by PEG/NaCl precipitation, and titrated as described in the NEB standard protocol to be used for the next round of selection. The two next rounds of selection were performed under more stringent conditions as the concentration of Tween-20 was gradually increased (0.3% and 0.5% for the second and third round, respectively) and the incubation time of phages with the target was gradually reduced (1.5 hours and 1 hour in the second and third rounds, respectively).

### Evaluation by ELISA of the phage-binding to B1 peptide

Fifteen phage clones were picked up randomly from the last round of biopanning, and were individually amplified and titrated. Approximately 1 × 10^10^ pfu of phages were added to each well pre-coated with the B1 peptide (100 µg) as previously described and incubated for 1 hour at room temperature and the unbound phages were removed with ten washes with 0.5% TBST. The anti-M13 monoclonal antibody (200 μL of dilution at 1:1000) were added, and incubated at room temperature for 1 hour with gentle agitation followed by incubation with horseradish peroxidase (HRP)-anti mouse for 1 hour. The bound antibody was visualized using 2,2′-Azino-bis (3-ethylbenzothiazoline-6-sulfonic acid) diammonium salt ABTS (from Sigma) and the absorbance was measured at OD 410 nm.

### Evaluation by ELISA of phage-binding to cell lines

HeLa/Gpt and HeLa/LMP1 (5 × 10^4^ cells/per well) were seeded into 96-well microplates and grown at 37 °C for 24 hours. Subsequently, cells were fixed with 4% paraformaldehyde in PBS and blocked with 5 mg/mL BSA in PBS for 1 hour at room temperature. After washing, 10^10^ pfu/well were added and incubated for 1 hour at room temperature with agitation. After washing with 0.5% TBST (10 × 2 minutes) the anti-M13 antibody (GE Healthcare) was added at 1:1000 and incubated for 1 hour at room temperature, followed by washing (6 × 2 minutes) with TBST before adding the anti-mouse antibody conjugated to the peroxydase (Vector Laboratories). The plate was incubated for 1 hour at room temperature, and then washed 6 × 2 minutes with TBST and ABTS (Sigma) was used to detect the bound antibodies as described previously.

### DNA Sequencing of selected phages

Positive phage clones giving the strongest ELISA signal were amplified in *E. coli* ER2738 strain and the M13 single stranded DNA was extracted according to the standard protocol. DNA sequencing was performed using the −96 gIII primer (5′-CCCTC ATAGTTAGCGTAACG-3′).

### Solid-phase peptide synthesis

The selected peptide B1.12 (ACPLDLRSPCG), as well as the first extracellular loop of LMP1: B1 (CMSDWTGG) were synthesized using the standard solid-phase Fmoc/tBu strategy on a Liberty Microwave-Enhanced Peptide Synthesizer (CEM GmbH, Germany), using hydroxybenzotriazole (HOBt) and 2-(1H-Benzotriazole-1-yl)-1,1,3,3-tetraméthyluronium (HBTU) as activators, and N,N-Diisopropylethylamine (DiPEA) as base in dimethylformamide (DMF). The Rink amide MBHA resin (100−200 mesh; 1.2 mmol/g, 1 g) was used as the solid-phase support, and the synthesis was performed on a scale of 20 μmol. The peptides were synthesized by consecutive deprotecting and coupling steps. Coupling step: Fmoc-protected amino acids (4 equivalents relative to resin loading), HBTU (0.5 M in DMF, 4 equiv), HOBt (0.5 M in DMF, 4 equiv), and DIPEA (2 M in DMF, 8 equiv). Deprotection: 20% piperidine in DMF (v/v). Side chain deprotection and cleavage of peptides from the resin were performed by treatment with trifluoroacetic acid TFA-TIS-EDT-H_2_O 95:-1-2-2 cocktail, at room temperature for 3 hours with agitation. After filtration of the resin, peptides were precipitated by addition of ice-cold ethyl ether, filtered, dissolved in water, and lyophilized. Peptides were then purified by RP-HPLC on a C18 XTerra semipreparative column (10 μm, 19 mm × 300 mm, Waters) with a linear gradient of water, 0.08% TFA (A)/acetonitrile, and 1% TFA (B) to a final purity of ≥95% and lyophilized. Characterization of purified peptides by Maldi-Tof analysis using MS/MS mass spectrometer Microflex LT Bruker was performed and purity and identity were assessed by HPLC analyses on a C18 XTerra (4.6 × 250 mm, 5 μm) column.

### NMR and ITC

The NMR experiments samples were prepared in 500 μL of H_2_O/D_2_O (90:10); using 1 mM of B1 peptide and 21 mM of B1.12 peptide. Titration experiment was also performed with varying the B1:B1.12 proportions (1:1; 3:1; 5:1; 7:1; 9:1). The NMR experiments were recorded on a Bruker spectrometer 500 MHz equipped with a TCI cryoprobe. Acquisition was performed with water suppression with excitation sculpting pulse sequence.

For ITC, analysis was performed at 30 °C using an isothermal titration calorimeter (VP-ITC, LLC Microcal Inc., Northampton, MA, USA). Peptides were suspended in NaHCO_3_ buffer (0.1 M, pH 5.7, after 2 hours of sample equilibration) and the reference cell was filled with the same buffer. B1 peptide (0.2 mM) was placed into 250 μL syringe and 1200 μL of B1.12 peptide (0.01 mM) was placed in the titration cell. The peptide solution was injected into the stirred sample cell containing B1.12 peptide in >20 injections (2 μL per injection) by a computer-controlled pump. The injection lasted 10 seconds with an interval of 120 seconds between successive injections. Heat released with each injection was measured (Differential Potential in µCal/sec) by the calorimeter in dynamic correction mode. Control experiments were performed by tittering the B1 peptide into the buffer solution at the same pH and concentrations with and without the B1 peptide, in order to discriminate the interaction process against the dilution of B1 and its possible self-association, respectively.

### Molecular docking

We constructed a three-dimensional model structure of the N-terminal region of the LMP1 protein based on the structure of the sweet transporter^29^ (PDB: 5ctgA), the Anabaena sensory rhodopsin^30^ (PDB: 1XIO), the *NE. Coli* YajR transporter^31^ (PDB: 3WDO) and the membrane domain of respiratory protein of *E. coli*^32^, (PDB:3RKO), by the use of GPCR-I-Tasser^33^. This module is a hybrid method designed for 3D prediction of trans-membrane proteins. The GPCR-I-Tasser pipline includes three steps, the first one consists of the generation of the trans-membrane helix, the second one consists of the I-Tasser fragment structure reassembly simulations and the third one consists of the selection of the model and the atomic refinement. Iterative Threading ASSEmbly Refinement server (I-TASSERcoupled to the GPCR) (http://zhanglab.ccmb.med.umich.edu/GPCR-I-TASSER/) template structures were selected by multiple threading approach Local Meta-Threading-Server (LOMETS) based on sequence similarity to the structure described at the top (Sweet transporter protein, the anabaena rhodopsin….). Alignments of the query sequence with each template were generated using the ConSurf server. This program provides multiple sequence alignment with conserved features, which provides information about specific amino acid features in their local environment. A total of five three-dimensional structure models were generated by GPCR-I-TASSER, among them, the best model was identified based on confidence score (C-Score) and the refined model coordinates were evaluated by MolProbity^34^. As the LMP1 atomic level structural data was not yet available, we constructed a model as described in our previous work^35^, and the B1.12 peptide model was generated similarly (all models are available as Supplementary Materials). To obtain a better understanding of the interaction between the N-terminal domain of LMP1 and the B1.12 peptide, we performed the modeling of the peptides binding to LMP1 in order to predict the positions and the orientation of the peptides in her binding cleft. We used a rigid protein-protein docking software packages that are based on different approaches, namely PatchDock^36^ and FireDock^37^. The PatchDock algorithm is inspired by object recognition and an image segmentation technique used in computer vision and applies geometric hashing and poseclustering matching to match concave and convex patches of interacting surfaces. A refined model of the complex using the NMR constraints was generated by patchDock. The web server is located at http://bioinfo3d.cs.tau.ac.il/FireDock/. FireDock transforms the model candidates generated by PatchDoc, and performs the refinement using a restricted interface side-chain optimization to allow a certain amount of steric clashes. The refined candidates are scored and ranked according to the energy function. A Final step of refinement was assessed with FG-MD^38^, which is a molecular dynamics algorithm for atomic-level protein structure refinement. FG-MD aims to refine the initial models closer to the native structure. It can also improve the local geometry of the structures by removing the steric clashes and improving the torsion angle and the hydrogen-binding networks. A final analysis with MolProbity was assessed to validate and to analyze the quality of the structures. Moreover, to better confirm the B1.12-LMP1 interaction, we have established two mutant models that are affected in the essential aa required for the interaction as indicated by NMR. As previously described, the Firedock software was used and the interaction of the different LMP1 mutants with the B1.12 peptide was studied.

### Competitive inhibition

Binding of the B1.12 peptide to its target B1 and to HeLa/LMP1 cells was evaluated by a competitive binding assay. For competition on B1 as a target, the B1.12 peptide or the unrelated control peptide were incubated at concentrations of 1, 5, 10, 25, 50 and 100 µM with B1 peptide (100 µl of a solution of 1 mg/mL in NaHCO_3_) at room temperature for 1 hour. Then the B1.12 phage (2 × 10^10^ pfu) was added to each well and incubated for 1 hour. After washing, the bound phage was detected by the anti-M13 antibody as described previously. For the competition test on HeLa cells expressing or not the viral oncoprotein LMP1, cells were seeded into 96-well plates (10^4^ cells/well), washed twice in PBS, and fixed with 4% paraformaldehyde for 30 minutes at room temperature. Synthetic peptides at concentrations of 50, 75, 100 and 200 µM were incubated with the cells at 37 °C for 1 hour and the experiment was carried out as described above. All experiments were done in triplicate and the mean values of OD were used to calculate the rate of competitive inhibition according to the following formula: inhibition ratio = (OD control − OD phage)/OD control × 100, where OD control and OD phage represent the OD450 nm values of phage binding on cells incubated with PBS and with the peptide, respectively.

### Cell viability assay

Cells were plated into 96-well plates until 80% confluent and then treated with different concentrations of B1.12 peptide (100, 200, 300 and 400 µM). After 48 hours of incubation, 20 μL MTT (5 mg/mL) were added to each well. After 4 hours, 100 μL dimethyl sulfoxide (DMSO) was added to each well, and absorbance was measured at 570 nm using a Varioskan™ Flash Multimode Reader (Thermofisher Scientific). The percentage of cell viability was calculated using this formula:(OD590 nm of treated cells/OD590 nm of untreated cells) × 100.

### *In vitro* cellular uptake by flow cytometry

The cellular uptake of the FITC labeled B1.12 peptide/unrelated peptide was analyzed by flow cytometry. C666.1, HeLa/LMP1, and HeLa/Gpt cells, were seeded at a density of 4 × 10^5^ cells/mL at 37 °C for 24 hours, and then incubated with 100 μM of both FITC-labeled peptides for 24 hours. Cells were washed three times, trypsinized, centrifuged at 1700 rpm for 5 minutes and then resuspended in 0.35 mL of PBS. The fluorescence associated to the cells was measured using LSR Fortessa X-20 (Becton Dickinson) for HeLa/LMP1 or Attune NxT Flow Cytometer (Thermofisher) cytometer for C666.1 and HeLa/Gpt cells.

### Fluorescence microscopy

To further investigate the fixation of the peptide on C666.1 cells, fluorescence microscopy was performed. Approximately 1 × 10^6^ cells were washed 3 times with PBS, fixed with 4% parafolmaldehyde for 15 minutes, blocked by 5 mg/mL BSA for 1 hour, and incubated with 10 μM FITC labeled B1.12 peptide or anti-LMP1 antibody (1/500 Dako) for 1 hour at room temperature. After being washed with PBS, the fixation of LMP1 was detected by Alexa-fluor 405 labeled secondary antibody (1/100). HeLa/Gpt cells were also stained as described above to serve as negative control. Images were analyzed using an EVOS Floid Cell Imaging Station (Life Technologies).

### Cell cycle analysis

Cell cycle analysis of C666.1 and HT29 cells was performed using the FXcycleTm PIi/RNnase staining solution (Invitrogen). Briefly, cells (4 × 10^5^) were seeded and grown for 24 hours, and then incubated with serum-free RPMI for another 24 hours (starvation) to decrease cell constitutive signaling. After starvation, cells were incubated or not with 50 µM B1.12 peptide (C666.1 and HT29 cells) or control peptide (C666.1 cells) for 48 hours. Following incubation, cells were washed with PBS, trypsinized and centrifuged at 1200 rpm for 5 minutes. The cell pellet was then washed twice with PBS, and cells were fixed with 5 mL of ethanol 70% for 24 hours at 4 °C. Aliquots of 2 × 10^5^ ethanol-fixed cells were then centrifuged for 5 minutes at 450 g, washed with PBS, and then resuspended in Propidium Iodide Reagent and incubated for 30 minutes at room temperature, shielded from light. The data were collected and analyzed by an Attune NxT Flow Cytometer (Thermofisher).

### Protein extraction and western blot

Cells were cultured at 80% confluency and then incubated with 100 µM B1.12 peptide for 24 hours. Subsequently, cells were washed twice with cold PBS and scrapped in ice-cold RIPA buffer (50 mM Tris pH 7.4, 5 mM EDTA pH 8.0, 150 mM NaCl, 0.1% SDS, 0.5% NaDOC, 0.5% NP 40) supplemented with 5 mM PMSF. After 30 minutes on ice, cells were sonicated, and cell debris were removed by centrifugation at 15,000 rpm at 4 °C for 15 minutes. Proteins were quantified using the Bradford Protein Assay (Bio-Rad) according to the manufacturer’s specifications. Thirty μg of total proteins were separated on 10% SDS–PAGE and transferred to PVDF membrane (Amersham Hybond-P). The membranes were blocked at 4 °C overnight with PBS 0.1% Tween 20 containing 5 mg/mL skimmed milk, prior to be incubated with pAkt (Biotechne, diluted 1/2500) pRelA536 (Biotechne, diluted 1/1000), and Tubulin (Thermofisher, diluted 1/1000) antibodies for 2 hours at room temperature, followed by incubation with anti-rabbit or anti-mouse conjugated to horseradish peroxidase at room temperature for 1 hour. The target protein band was visualized using the enhanced chemiluminescence detection system (Amersham ECL Prime Western Blotting Detection Reagent) and luminescent bands were visualized using the VERSADOC imaging system. Band densities were assessed by Image-J software.

### cDNA synthesis and RT-qPCR analysis

Total RNA from C666.1 cells treated or not with B1.12 and control peptides was extracted using TRIzol reagent. cDNA was synthetized using the reverse transcription kit (Invitrogen), and served as a template for detecting A20 gene expression by Real-time quantitative PCR using SYBR Premix Ex Taq (Tli RNase H Plus, Takara) and using CFX96 (Biorad). The housekeeping gene GAPDH was used as an internal control to normalize the expression levels of the gene. The primers used for the amplification of the A20 gene are as follows: forward: 5′-GCCCGAAGTGGACTTCAGTACAAC-3′, and reverse: 5′-GGCGAAATTGGAACCTGA TTCCAAACTT-3′. Relative quantification of mRNA expression was performed by the comparative 2−ΔΔct method to calculate the difference between amplifications of A20 and GAPDH. The experiments were performed with three technical replicates, and the mean fold changes and standard deviation were also calculated.

### Statistical analysis

Statistical analysis was performed by GraphPad™ Prism 5.0 software (Graphpad Software Inc., San Diego, CA, USA) and values were considered significant when P < 0.05.

## Supplementary information

Supplementary materials

## Data Availability

The data that support the finding of this study are available from the corresponding author upon request.
