# Supplementary material for: B1.12: a novel peptide interacting with the extracellular loop of the EBV oncoprotein LMP1
Source: Sci Rep. 2019 Mar 13;9:4389. doi: 10.1038/s41598-019-39732-y (PMC6416395; doi:10.1038/s41598-019-39732-y)
Supplement: Supplementary file 1 — Supplementary materials [file 41598_2019_39732_MOESM1_ESM.pdf]

B1.12: a novel peptide interacting with the extracellular loop of the EBV oncoprotein LMP1.

Nihel Ammous-Boukhris[1], Amor Mosbah[2], Wajdi Ayadi[1], Emna Sahli[5], Soizic Chevance[3], Arnaud Bondon[3,4] , Ali Gargouri[1], Michele Baudy-Floc'h[3] , and Raja Mokdad-Gargouri[1]*

Supplementary Materials 1

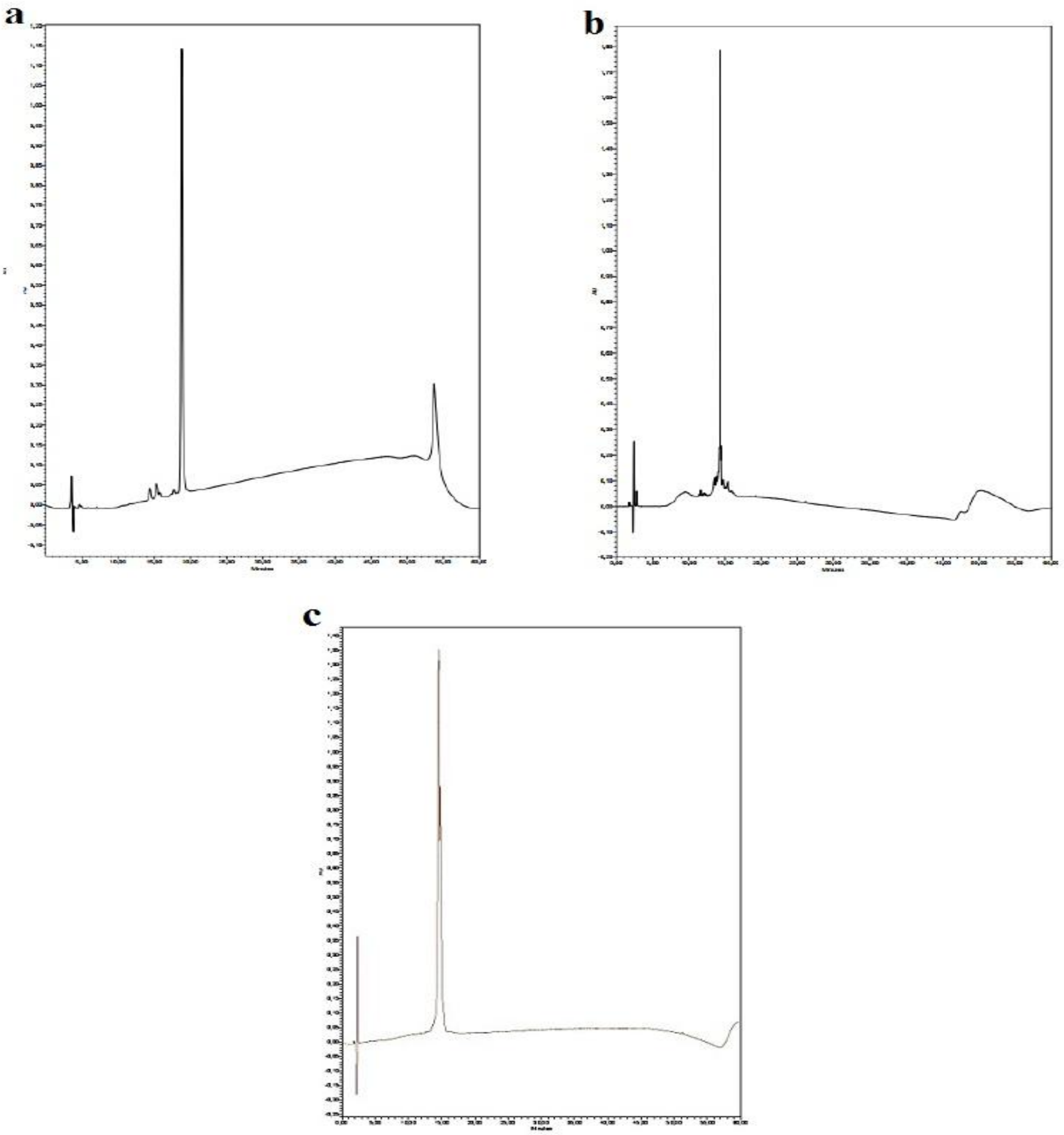

Figure S1: Chromatograms showing the purity of synthesized peptides (a) B1, (b) B1.12, and (c) B4.

B1.12: a novel peptide interacting with the extracellular loop of the EBV oncoprotein LMP1.

Nihel Ammous-Boukhris[1], Amor Mosbah[2], Wajdi Ayadi[1], Emna Sahli[5], Soizic Chevance[3], Arnaud Bondon[3,4] , Ali Gargouri[1], Michele Baudy-Floc'h[3] , and Raja Mokdad-Gargouri[1]*

Supplementary Materials 2

1 2 3 4 5 6

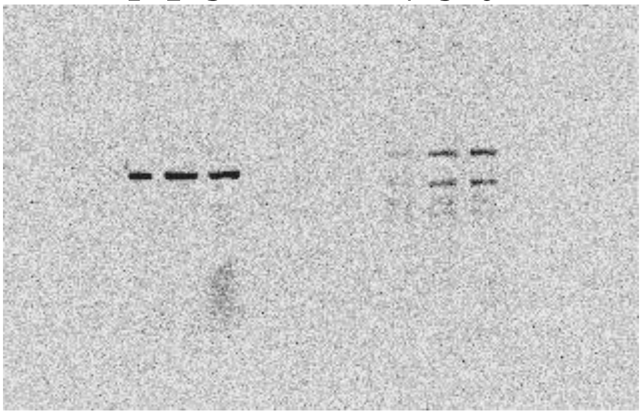

Figure S2: Western blot analysis of pNFκB (1, 2, and 3) and pAkt (4, 5, and 6) expression, in C666.1 cell line treated (1, 4) or not (2, 5) with 100 µM of B1.12 or with 100 µM of a control peptide (3, 6).

1 2 3 4 5 6

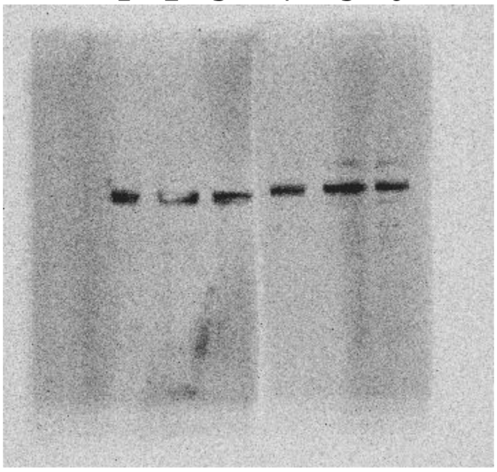

Figure S3: Western blot analysis of tubulin expression, in C666.1 cell line treated (1, 4) or not (2, 5) with 100 µM of B1.12 or with 100 µM of a control peptide (3, 6).
